# Cognitive flexibility moderates the association between maltreatment and emotion regulation in residential care children of the middle childhood period

**DOI:** 10.1177/25161032221100233

**Published:** 2022-05-06

**Authors:** Laetitia Mélissande Amédée, Laurence Cyr-Desautels, Houria Bénard, Katherine Pascuzzo, Karine Dubois-Comtois, Martine Hébert, Célia Matte-Gagné, Chantal Cyr

**Affiliations:** 1 14845 Université du Québec à Montréal, Canada; 2 14847 Université du Québec à Trois-Rivières, Canada; 3 Institut Universitaire Jeunes en Difficulté, 49987 CIUSSS du Centre-Sud-de-l’Île-de-Montréal, Canada; 4 7321 Université de Sherbrooke, Canada; 5 Hôpital en Santé Mentale Albert-Prévost, 439501 CIUSSS du Nord-de-l’Île-de-Montréal, Canada; 6 4440 Université Laval, Canada

**Keywords:** Residential care, child maltreatment, executive functions, emotion regulation, cognitive flexibility

## Abstract

The purpose of this study was to examine, in a sample of residential care children, the moderating role of cognitive flexibility in the association between maltreatment and emotion regulation competencies. The sample included 69 children aged 8 to 12 and their group home educator as their primary caretaker. Educators completed questionnaires evaluating child emotion regulation competencies and cognitive flexibility. Child history of maltreatment and sociodemographic data were collected on the basis of the children’s child protective services files. Moderation analyses showed that the effect of child maltreatment on emotion regulation was only present for children with high levels of cognitive flexibility, such that the less children experienced severe maltreatment, the more they showed emotion regulation competencies. Children with low cognitive flexibility displayed lower levels of emotion regulation regardless of their maltreatment history. These results suggest that focusing on cognitive flexibility when intervening with children in residential care could help strengthen their emotion regulation competencies, which may prevent further maladaptive behaviors.

Child maltreatment is a pervasive public health problem associated with cognitive, psychological, and behavioral difficulties (Toth & Manly, 2019). In 2018, there were 678,000 reports of child maltreatment in the United States and 10% of these children were placed in group or residential care (Child Welfare Information Gateway, 2020; U.S. Department of Health et Human Services, 2020). This proportion is similar in Canada and in Québec (Directeurs de la protection de la jeunesse, 2021; Fallon et al., 2020) where the current study was conducted. Research has shown that children in residential care, compared to other types of out-of-home placement (kinship care, foster care), are at a greater risk of psychosocial maladaptation (Briggs et al., 2012; Li et al., 2019). These children often experience multiple, more severe and chronic forms of maltreatment and show high levels of psychological dysfunction (Collin-Vézina et al., 2011; Cyr et al., 2012). The high proportion of children in residential care displaying psychosocial difficulties can be explained, in part, by the cumulative effect of maltreatment. In fact, studies in the field of maltreatment have found children that who have experienced both repeated and diverse forms of maltreatment are more at risk of maladaptive behaviors and psychological problems (Contractor et al., 2018; Finkelhor et al., 2007; Warmingham et al., 2019)

Although research about the neurocognitive outcomes of maltreatment is emerging, there is a paucity of studies on children living in residential care, especially in the middle childhood period (Hodgdon et al., 2018; Moreno-Manso et al., 2019). Information on the cognitive and emotional regulatory processes of these children seems rather important, given that these processes are generally targeted in care treatment programs aiming at children’s rehabilitation (Centre jeunesse de Montréal-Institut universitaire, 2011). Hence, the present study investigated whether cognitive regulatory processes explain the expected association found between severity of maltreatment and emotion regulation competency in residential care children of the middle childhood period.

## Child maltreatment and emotion regulation

Child maltreatment is generally defined as the occurrence of physical, emotional, or sexual abuse as well as neglect (Cicchetti & Valentino, 2006). Research in the last decade has strayed from studying single types of maltreatment to instead focusing on the effect of multiple types on children’s functioning because of the high rates of cooccurrence of maltreatment types (Contractor et al., 2018; Finkelhor et al., 2007; Petruccelli et al., 2019). For example, a recent study among maltreated and non-maltreated children found that 57% of the maltreatment group had experienced multiple subtypes of maltreatment (physical, sexual, emotional abuse, or neglect) across multiple developmental periods (Warmingham et al., 2019). The idea that cumulative adverse experiences, including experiences of maltreatment, worsen outcomes has been pioneered by the ACE study, which found that having endured multiple types of adverse childhood events (i.e. child abuse and neglect and family dysfunction) was associated with poorer health outcomes (Felitti et al., 1998). Finkelhor and colleagues have referred to this phenomenon as poly-victimization, which they defined as having endured four or more types of interpersonal and community violence during childhood (Finkelhor et al., 2007). Other researchers have referred to cumulative childhood trauma to reflect having experienced multiple types of maltreatment such as sexual, physical, or emotional abuse and exposure to interparental violence and neglect (Hodges et al., 2013). While authors have used different terms to define the cumulative effect of trauma, they all found that the diversity, multiple, and/or chronicity across time of traumatic experiences were associated with more psychological difficulties in childhood (Contractor et al., 2018; Finkelhor et al., 2007; Warmingham et al., 2019). In the present study, the term cumulative maltreatment is used to reflect both the reoccurrence of maltreatment episodes and having experienced multiple types of maltreatment.

The association between child maltreatment and emotion regulation difficulties is well established. A recent meta-analysis of 35 studies with children and adolescents (5 to 18°years old) who have experienced child maltreatment (defined as physical abuse, sexual abuse, emotional abuse, physical neglect, and/or emotional neglect) showed a significant negative association, with medium effect size, between child maltreatment and emotion regulation competencies (Gruhn & Compas, 2020). Studies have also found a significant association between cumulative trauma and emotion regulation. Two studies conducted with school-aged children who have experienced child sexual abuse found that experiencing other co-occurring forms of maltreatment (physical abuse, psychological abuse, exposure to family violence, traumatic separation, and/or neglect) was associated with less emotion regulation competencies and more emotion dysregulation (Choi & Oh, 2014; Hébert et al., 2018). Another study among children aged 10 to 12 found that children who experienced chronic maltreatment and were exposed to multiple types of maltreatment had more difficulty regulating their emotions than children who had experienced a single type of maltreatment (Warmingham et al., 2019). Similar results have been found among young adults with a history of childhood maltreatment (Warmingham et al., 2022). Findings thus suggest that maltreated children are at risk of experiencing emotion regulation difficulties, no matter the type or cumulative nature of their experienced maltreatment.

Emotion regulation competencies are the ability to understand, process, and display appropriate emotions (Cicchetti et al., 1995). These cognitive and behavioral processes not only have biological foundations but are also learned through socialization (Stålnacke et al., 2019). From early childhood, the primary caregiver, via day-to-day interactions, promotes the child’s development of emotional understanding and coping strategies (Thompson & Goodman, 2010; Meuwissen & Carlson, 2019). However, when parental trust is broken because of abuse or neglect, parents lose their role as external regulators (Speidel et al., 2019). As a result, children’s difficulties in regulating emotions increase, which in turn may lead to the display of maladaptive behaviors (Kim-Spoon et al., 2013). Nevertheless, not all maltreated children show problems in regulating emotions, and some studies found that emotion regulation competencies among maltreated children predict psychosocial adaptation of these children (Kim & Cicchetti, 2010; Rodman et al., 2019). This suggests that risk and protective factors may moderate the relation between child maltreatment and emotion regulation.

## Child maltreatment and executive functioning

Research regarding the association between child maltreatment and executive functioning is emerging. Executive functions are higher-order cognitive functions responsible for regulating goal-directed actions. The main executive functions are working memory, inhibitory control and cognitive flexibility (Diamond, 2013). Although some studies have conceptualized executive functioning as a single concept, authors have stressed the importance of investigating different components of executive functioning as they might have differentiated effects on child adaptation (Augusti & Melinder, 2013; Friedman & Miyake, 2017; Karr et al., 2022). While some studies have found that child maltreatment was associated with poorer performance on executive functioning tasks as well as greater self-reported executive functioning difficulties, other studies have found no such associations. For instance, a study among preschoolers found a significant association between child maltreatment and poorer performance in inhibitory control and cognitive flexibility tasks (Fay-Stammbach & Hawes, 2019). Another study comparing children aged 8 to 12 who had experienced physical abuse, neglect or witnessed intimate partner violence to control participants found no group difference in inhibitory control and cognitive flexibility (Augusti & Melinder, 2013). Given these inconsistent findings, executive functioning may be a potential moderator between child maltreatment and emotion regulation competencies. For example, a study found that executive functioning moderated the association between poly-victimization (defined as the number of maltreatment types experienced) and externalizing behaviors in preschoolers living in foster care (Horn et al., 2018).

## Child maltreatment, cognitive flexibility and emotion regulation

An important dimension of executive functioning most malleable by the environment is Cognitive flexibility (Rosen et al., 2020). Cognitive flexibility refers to the ability to adjust one’s behavior quickly, adaptively, and according to the changes in the environment (Amorim & Marques, 2018). Cognitive flexibility starts developing during preschool years and significantly expands during middle childhood (Buttelmann & Karbach, 2017). This suggests that during this developmental period, cognitive flexibility is more sensitive to influences of the environment (Lucassen et al., 2015; Müller & Kerns, 2015; Wiebe & Karbach, 2017). Child maltreatment has been associated with poor cognitive flexibility in middle childhood and adolescence (Nolin & Ethier, 2007; Spann et al., 2012). Namely, a study among children aged 8 to 12 years old, with and without a history of maltreatment, reported that maltreated children performed more poorly in a cognitive flexibility task (Carvalho et al., 2020). Another study among children aged 6 to 12 showed that maltreated children displayed more difficulty in cognitive flexibility than those in the community control group (Dileo et al., 2017). Nonetheless, a comparative study conducted among preschoolers showed that only a third of maltreated children displayed clinically significant levels of cognitive flexibility deficits, suggesting variability in the effect of child maltreatment on cognitive flexibility (Fay-Stammbach & Hawes, 2019).

It has been posited that through positive relational experiences, such as responsive caregiving, children could develop more cognitive flexibility (Curran & Andersen, 2017; Mills-Koonce et al., 2016). A study among a normative sample of preschoolers and their primary caregiver showed mother’s emotional support was longitudinally associated with greater flexibility in children (Zeytinoglu et al., 2019). For children in placement, such as residential care children who may have limited contact with their parents, a positive and healthy relationship with a new caretaker may facilitate the development of cognitive flexibility. Indeed, when placed in residential care, children have the opportunity to develop a significant relationship with an educator who spends time with them on a daily basis, talks about personal matters, and through their active presence, becomes a meaningful caregiving figure that may help with the growth of cognitive skills (Chartrand et al., 2013). In support of the hypothesis that healthy relationships with significant adults promote cognitive flexibility are the results of an intervention study with foster-care children and their parents. Findings revealed that children aged four to 6 years who were exposed to more positive parental behavior following a short parent-child intervention aiming to promote parental sensitivity and child attachment security showed higher levels of cognitive flexibility at the end of the intervention in comparison to children of the control group (Lewis-Morrarty et al., 2012).

Cognitive flexibility also plays a role in children’s ability to regulate emotion (Happaney et al., 2004). For example, deficits in cognitive flexibility have been associated with rigid stress management strategies, such as avoidance symptoms in adolescents placed in residential care (Hodgdon et al., 2018). Conversely, higher cognitive flexibility has been found to predict greater emotion understanding. More specifically, they were better at recognizing and responding to their emotion and those of others among normative preschoolers (Wang et al., 2021). However, there is a paucity of research about the association between cognitive flexibility and emotion regulation competencies in maltreated children. Nevertheless, findings support a resiliency model in which cognitive flexibility would act as a protective factor against the negative impacts of early life adversity. This could be particularly the case for children in residential care for whom cognitive flexibility may be improved via the relationship with new caregivers and interactions with peers. In such a moderation model, emotion regulation competencies would not (or to a low degree) be associated with the cumulative maltreatment in children with higher levels of cognitive flexibility, but it would for children with lower levels of cognitive flexibility.

## The present study

Considering the literature supporting the associations between experienced maltreatment, difficulties in cognitive flexibility, and poorer emotion regulation among vulnerable children, understanding the interactive association of child maltreatment and cognitive flexibility with children’s emotion regulation competencies could shed light on relevant intervention targets for children in residential care. To date, no study has examined the association between cumulative child maltreatment, cognitive flexibility, and emotion regulation among school-aged children in residential care. In addition, middle childhood is a crucial period in the development of both cognitive flexibility and emotion regulation competencies through brain maturation and increased socialization with significant others, such as peers and other caregivers (Valiente et al., 2020; van Lier & Deater-Deckard, 2016). Against this backdrop, the objective of this study is to examine the associations between cumulative maltreatment, cognitive flexibility, and emotion regulation among residential care children between the ages of 8 and 12. Specifically, we examine whether the association between cumulative maltreatment and emotion regulation competencies is moderated by cognitive flexibility.

## Method

### Participants and procedures

Data from this study come from a larger ongoing study investigating the adaptation of 69 children living in residential care during the middle childhood period. The current study reports on data provided by questionnaires administered to children’s main educators (81.2% women) who assumed the role of primary professional caregivers. Residential care children (71.0% boys) had a mean age of 9.57 (*SD* = 1.45). Children and their educators were recruited in urban cities in the province of Québec, Canada. To be included in the larger project, children had to be (1) aged between 8 and 12 years old, (2) placed in a residential treatment center or community group home for the last 6 months, and (3) assigned to the same educator for the past 6 months. Community group homes are houses usually situated in a residential area that can host a small group of children, while residential treatment centers are situated in government buildings where children are divided into small groups and receive multidisciplinary care. Biological parents of the children were first contacted to obtain a signed consent for their child’s participation in the project. Children for whom we obtained parental consent were then contacted for their assent. Consent forms were also signed by the educators for their own participation in the study. Educators’ sex was documented at that moment. Trained doctoral-level research assistants met the children and their educators at their residency twice within a 4-week period. Questionnaires were given to educators on the first visit and returned to the research team on the second visit. Ethical approval for this study was granted by the Ethics committees of the Centre Intégré Universitaire de santé et de services sociaux du Centre-Sud-de-l’île-de-Montréal.

### Measures

***Executive functions*.** Educators completed the Behavior Rating Inventory of Executive Function (BRIEF; Gioia, Isquith, Guy, Kenworthy et Ptáček, 2011), evaluating children’s executive functioning. In this study, the subscale assessing cognitive flexibility was used. This subscale is composed of seven items rated on a 3-point Likert scale (1 = never to 3 = always). A higher score indicates cognitive flexibility deficits. The clinical cut-off score for the cognitive flexibility scale is a T-score of 64. This scale has shown convergent validity with observational measures of executive functioning during middle childhood (Carrera, Jiménez-Morago, Román et León, 2019). Studies have found higher T-scores in maltreated children compared to those in normative samples (Dileo et al., 2017; Fay-Stammbach et Hawes, 2019). The internal consistency of this scale for the present sample was .80.

***Emotion regulation*.** Emotion regulation was evaluated with the Emotion Regulation Checklist, which assesses emotion regulation competencies and dysregulation (ERC; Shields & Cicchetti, 1997). Professional caregivers answered 24 questions on a 4-point Likert scale (1 = never to 4 = almost always). In this study, the emotion regulation competency subscale was used, for which a higher score indicates more appropriate affective displays (range of 1 and 32). Studies among maltreated children have found higher T-scores in maltreated children than in normative samples (Blandon et al., 2010; Kim-Spoon et al., 2013; Shipman et al., 2005, 2007). The internal consistency of the scale for this sample was .75.

***Children’s Child Protective Services (CPS) files*.** Children’s sociodemographic information (child age, sex, and ethnicity) and their history of maltreatment (number of CPS reports, types of maltreatment) and out-of-home placements (quantity and length) since birth were collected from CPS files.

Child maltreatment was legally assessed by CPS practitioners according to legal definitions of maltreatment in Quebec (Loi sur la protection de la jeunesse, 2021): a) sexual abuse, serious risk of sexual abuse, or attempted sexual contact between a caregiver (or responsible adult) and a child; b) physical abuse: injuries inflicted by an adult on a child by non-accidental means; c) neglect: failure to provide minimum standards of physical care; and d) emotional maltreatment: failure to provide for psychological safety, security or basic emotional needs. Cases could be reported to CPS for one of the four types of maltreatment or for being at serious risk of being maltreated. As prior research has shown, there is no difference in outcomes between substantiated and unsubstantiated CPS reports; both types of reports were computed in that score (Kugler et al., 2019).

Finally, a cumulative maltreatment score was computed by adding the number of CPS reports and the number of maltreatment types children have sustained. Reflecting both the concept of revictimizations (the same type of maltreatment on multiple occasions) and cumulative trauma (multiple types of maltreatment). This method is supported by research that suggests a cumulative effect of the number and diversity of traumatic events on psychological adjustment (Contractor et al., 2018; Finkelhor et al., 2007; Warmingham et al., 2019). For example, a child who was reported three times to CPS for two different types of maltreatment (e.g., physical abuse and neglect) would have a score of 5, whereas a child who was reported three times for the same type of maltreatment (e.g., physical abuse) would obtain a score of 4.

### Data analysis plan

First, we describe the sample in terms of sociodemographic variables and children’s maltreatment and placement history. Mean scores for the study variables (emotion regulation and cognitive flexibility) are also presented.

Second, we conducted preliminary analyses, such as correlations and *t*-tests, to examine potential covariates (child placement history, child sociodemographic characteristics, educators’ characteristics) with child emotion regulation, the dependent variable, and cognitive flexibility as the moderator. Finally, correlational analyses were conducted to examine the intercorrelations between study variables (cumulative maltreatment, emotion regulation, cognitive flexibility).

Moderation regressions, as main analyses, were performed with the SPSS *Process Macro,* using multiple regressions with bootstrap routines. To avoid potential multicollinearity between predictors, the cumulative maltreatment continuous score and the cognitive flexibility variable were mean-centered prior to computing the interaction term.

## Results

### Descriptive statistics

***Child and caregiver characteristics*.** Table 1 shows descriptive statistics for study variables. Most children were of French Canadian descent (81.1%), followed by Haitian (12.2%), and Arabic, Latin or unspecified origin (6.7%). Information from children’s CPS files revealed that since birth, 81% of children had experienced neglect, 43% physical abuse, 12% sexual abuse, and 36% emotional maltreatment. Most children (62%) had experienced at least two different forms of child maltreatment. Children were reported to CPS an average of 5.19 times (*SD* = 3.47; range = 1 and 18). They had experienced nearly 4 (*M* = 3.91, *SD* = 2.72; range 1 and 15) out-of-home placements, including residential care and foster-family placements. As for their length of time spent in placement since birth, the average was 44.62 months for the current sample (range = 6.87 and 124.33). Children obtained a mean cumulative maltreatment score of 6.83 (SD=3.73; range = 2 and 21).

**Table 1.** Descriptive statistics and correlations for study variables.

Variable	*Range*	*M*	*SD*	1	2
1. Cumulative maltreatment	2–21	6.83	3.73	—	
2. Cognitive flexibility deficits	39–85	62.38	10.99	−.25*	—
3. Emotion regulation	10–29	21.00	3.50	−.14	−.31**

*p < .05; **p < .01.

At intake, children were placed in two types of residential placement, 49.3% of the children were residing in community group homes and 50.7% in a residential treatment center. Children were in their current placement for an average of 20.26 months (range = 6 to 58). We examined whether child age (*t* = −1.52; *p* = .13) and sex (*χ*^*2*^ = 0.05; *p* = .82) varied as function of types of placement (residential treatment centers vs. community group homes). No significant differences were found. As for caregivers, most were women (81.2%).

Descriptive statistics also indicated that the average score on the cognitive flexibility deficit scale was 62.38 (*SD* = 10.99; range = 39 and 85). Based on the cut-off score of cognitive flexibility, 39.1% of the sample reached clinical levels. The average score on the emotion regulation scale was 14.99 (*SD* = 3.50; range = 4 and 23).

### Preliminary analyses

Correlations and *t*-tests were conducted to examine whether sociodemographic data (i.e. child age, gender, ethnicity), placement characteristics (number, length, and type), educator’s characteristics (gender), as potential covariates, were related to child emotion regulation, as the dependent variable. No significant associations were found for child age (*r* = −.08, *p* = .51), gender (*r* = −.01, *p* = .94), ethnicity (*r* = .15, *p* = .24), number (*r* = .20, *p* = .09), length (*r* = .11, *p* = .38), type (*t* = −1.49, *p* = .94) of placement and educator’s gender (*t* = −0.22, *p* = .84). Given that these variables were not related to the dependent variable, no covariates were included in the main analyses.

Furthermore, we examined if sociodemographic data (i.e., child age, gender and ethnicity), placement characteristics (length, number, and type), and educator’s characteristics (gender) were related to child cognitive flexibility. No significant association was found for child age (*r* = .10, *p* = .11) and ethnicity (*r* = −.23, *p* = .65), number of placements (*r* = .18, *p* = .14) and educator’s gender (*t* = 0.34, *p* = .74). There was a significant difference between boys (*M* = 64.18, *SD* = 11.36) and girls (*M* = 57.95, *SD* = 8.79) on the cognitive flexibility deficits scale (*t* = −2.45, *p* = .02). Boys had more cognitive flexibility deficits than girls. Children in residential treatment centers (*M* = 65.03, *SD* = 9.46) had more cognitive flexibility deficits (*t* = −2.23, *p* = .03) than those in community group homes (*M* = 59.27, *SD* = 11.06). A correlation between length of placement and cognitive flexibility deficits was also significant (*r* = .30, *p* = .01), indicating that children who were placed for a longer period of time showed more cognitive flexibility deficits.

Finally, intercorrelations among study main variables (cumulative maltreatment score, emotion regulation competencies, and cognitive flexibility) were examined. The cumulative maltreatment score was significantly correlated with cognitive flexibility deficits (*r* = .25, *p* = .04), but not with emotion regulation competencies (*r* = −.13, *p* = .28). A significant correlation was also found between cognitive flexibility deficits and emotion regulation (*r* = −.31, *p* = .01).

### Main analyses: Moderation model

**Figure 1.**
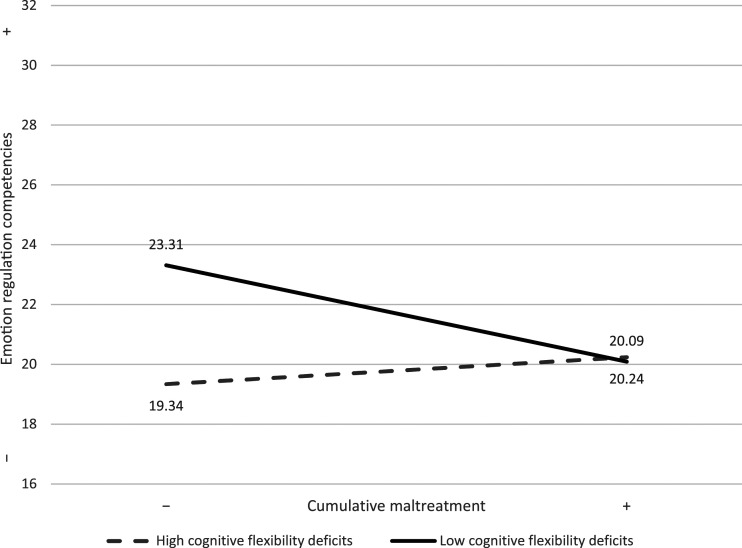
Cognitive flexibility as a moderator between child maltreatment and emotion regulation. *Note.* High cognitive flexibility deficits = poorer functioning; Low cognitive flexibility deficits = adaptative functioning.

A regression analysis tested whether the level of maltreatment severity and level of cognitive flexibility deficit and their interaction term (Maltreatment severity X Cognitive flexibility deficit) were associated with emotion regulation competencies. Statistics for model summary, including unstandardized beta coefficients and standard errors (S.E.) for each effect, are reported with 95% confidence intervals (CI) and presented in Table 2. The model summary for the regression was significant, *F*(3, 65) = 4.23, *p* = .009, and explained a total variance of 16%. The results showed no significant main effect of maltreatment severity on emotion regulation, *b* = −0.58, S.E. = 0.44, *p* = .20. However, less cognitive flexibility deficit was significantly associated with a higher quality of emotion regulation, *b* = −0.90, S.E. = 0.04, *p* = .02. The interaction term was also significant, *b* = .09, S.E. = 0.04, *p* = .03, and explained 6.3% of the total variance. This result indicates that for children with less cognitive flexibility deficits (higher cognitive flexibility), the less they cumulated experiences of maltreatment, the more they showed emotion regulation competencies, *b* = −1.61, S.E. = 0.76, CI [-3.13 ∼ −0.10]. The slope for children with more cognitive flexibility deficits indicated no significant relation between maltreatment severity and emotion regulation, *b* = 0.45, S.E. = 0.51, CI [-0.56–1.46]. Figure 1 illustrates the slopes for children with more and less cognitive flexibility deficits.

**Table 2.** Hierarchical regression analyses predicting emotion regulation.

Predictor variables	Δ*R*^2^	*F*	df	*b*	95% *CI*
Emotion regulation competencies					
Step 1		4.23	(3,65)		
Maltreatment severity				−.58	(−1.4695 ∼ .3073)
Cognitive flexibility deficits				−.09*	(−.1619 ∼ −.0120)
Maltreatment severity × cognitive flexibility deficits	.06	4.90	(1,65)	.09*	(.0091 ∼ –.1785)

*Note.* Emotion regulation competencies: Total *R*^2^ = .16; *p < .05.

## Discussion

It is well-established that child maltreatment has pervasive effects on children’s development (Cicchetti et Valentino, 2006). Understanding the mechanisms under which these problems develop helps inform on the protective factors that are essential to the implementation of personalized intervention programs. The purpose of this study was to examine, in a sample of residential care children between the ages of 8 and 12, the potential moderating role of cognitive flexibility in the association between maltreatment severity and emotion regulation competencies.

First, on a descriptive level, we found that 39.1% of children reached the clinical threshold for cognitive flexibility problems. Although not directly comparable, this result is in line with a study on maltreated preschoolers (4-5 years old) that found 31% of the children had clinical scores of cognitive flexibility problems (Fay-Stammbach & Hawes, 2019). These results suggest that although child maltreatment is associated with less cognitive flexibility, the majority of children will not display clinical levels of cognitive flexibility problems. This could perhaps be due to good relational experiences with other caregivers, such as an educator in the residential care setting (Curran & Andersen, 2017).

Second, in our sample, children displayed lower emotion regulation competencies compared to studies with non-maltreated children. This is consistent with other studies indicating lower scores of emotion regulation competencies among non-institutionalized maltreated children compared to community controls (Kim-Spoon et al., 2013; Shipman et al., 2007). In Quebec, residential care is usually the last resort for children in need of out-of-home care arrangements. Placement in community group homes or residential treatment centers comes when other types of placements do not make it possible to respond to the multiple and/or severe difficulties of the children. These difficulties could be explained by a developmental history of high adversity, which could include an accumulation of forms of maltreatment. As shown by our results, children from our sample have experienced multiple out-of-home placements for a long proportion of their life, considering that children were on average nine and a half years old and have been placed under out-of-home care for an average of 6 years.

Findings of our preliminary analyses show that cumulative maltreatment was not associated with emotion regulation competencies in the present study. Although this finding could be due to the lack of variability in our sample, as most children displayed low emotion regulation skills, it could also indicate that in high-risk samples, the association between cumulative maltreatment and emotion regulation could be moderated by potential protective factors that render some maltreated children resilient in the face of adversity or on the contrary, contingent on factors increasing the risk of showing problems. As we argued earlier, among moderators that may be at play here are those related to the children’s personal resources, such as cognitive flexibility. A prior study had found for children placed in foster care that poly-victimization was only associated with externalizing symptoms when children displayed low executive functioning (Horn et al., 2018). Another found that maltreated children with high executive functioning were more resilient and displayed lower levels of externalizing behaviors compared to children with low executive functioning (Obradović, 2010).

Results from the moderation analysis conducted in this study supported the hypothesis that cognitive flexibility moderates the association between cumulative maltreatment and emotion regulation competencies. However, the results of our study did not show the expected relation that more cognitive flexibility protected those with more severe maltreatment experiences. Our findings indicate that children who have experienced less maltreatment and are able to display more cognitive flexibility will find it easier to regulate themselves in comparison to those with lower cognitive flexibility and to those with both higher cognitive flexibility and more severe experiences of maltreatment. Precisely, when children display good cognitive flexibility, they are more attuned to their environment, and the accumulation of maltreatment experiences could affect them more (or less if they have experienced less maltreatment).

As for children with deficits in cognitive flexibility, the effect of maltreatment on emotion regulation was not present, which is not in line with past studies generally showing that cognitive flexibility deficits are associated with less ability to adjust to stressful situations adequately and regulate emotions (Happaney et al., 2004; McClelland & Cameron, 2012). The results of our study may be explained by the small size of the sample affecting statistical power as well as the lack of variability (more children with lower scores of cognitive flexibility deficits) on the cognitive flexibility scale. Another explanation could be that when children have cognitive flexibility deficits, they do not benefit as much from their environment and therefore are less impacted by their maltreatment history. On the one hand, children with more cognitive flexibility deficits could be, in a certain way, protected from serious episodes of abuse, but on the other hand, when they have experienced less abuse, it could still be difficult for them to adapt.

Although this result does not entirely support the protective role of cognitive flexibility for children with more severe early life adversity, we need to acknowledge that the children of our sample with less severe adversity still have experienced a fair amount of maltreatment experiences early in life. In addition, it is to be noted that although these less severely maltreated children with more cognitive flexibility showed higher emotion regulation than their peers. Their mean score was lower (*M* = 22.47) than that found for children of normative samples (equal or higher than = 25) (Blandon et al., 2010; Kim-Spoon et al., 2013; Shipman et al., 2005, 2007). Consequently, because the children of our sample with less severe experiences of maltreatment were still highly exposed to maltreatment, we can conclude that cognitive flexibility is a protective factor against the development of emotion regulation difficulties. Nevertheless, our results may mean that working on improving children’s emotion regulation through cognitive flexibility is probably more beneficial for those who have experienced less severe experiences of maltreatment.

### Strengths and limitations

To our knowledge, this study is the first to examine cognitive flexibility as a potential moderator of the association between child maltreatment and emotion regulation in residential care children of the school-age period. Although the final size of our sample is small, limiting the statistical power of our study, the number of children in our sample is substantial, given that many published studies have relied on very small samples (e.g., Collin-Vézina et al., 2011; Shechory & Sommerfeld, 2007) and that conducting research with the Child Protective System is filled with obstacles. For example, parental consent, an essential research step, is not easy to obtain when most parents are difficult to reach, even with the help of the children’s educators. Hence, our study is a first step in understanding the emotional and cognitive functioning of children living in residential care*.* Another strength of this study is that only primary professional caregivers of children were asked to fill out questionnaires about children that were under their care for at least the last 6 months. Residential care children are exposed to many educators but are often assigned a principal educator that acts as the primary caregiver and is primarily responsible for the child’s intervention plan.

Despite these strengths, our study relies on the use of only one informant and a cross-sectional design, which may have created shared-method variance. However, our study uses information from children’s CPS files which provides a retrospective measure of maltreatment. We also were not able to gather more information about educator characteristics. We encourage researchers to implement longitudinal research designs using a multi-informant approach to examine children’s psychosocial development throughout their stay in residential care to obtain a more thorough understanding of the impact of maltreatment on their later functioning. This design is even more important to consider given that child maltreatment can significantly disrupt the development of the brain (Daly et al., 2017) and that cumulative maltreatment may have lifelong biopsychosocial impacts (Charak et al., 2016). Finally, future studies should also consider assessing executive functioning with observational, cognitive tasks to avoid perception bias of the informants (Monette & Bigras, 2008).

### Clinical implications

The results of this study show the importance of proper evaluation of children’s emotion regulation competencies as well as executive functioning at intake, as many children will display difficulties in both cognitive flexibility and emotion regulation, and these skills are associated with children’s functioning and rehabilitation. They also support the implementation of programs such as the Attachment, Self-Regulation and Competency framework (Brend et al., 2020). This program was developed for poly-victimized children and their caregivers (parents and educators) and is currently being implemented in Quebec Child Protection Residential Care units (Brend et al., 2020). Because this program is designed in a modular manner, activities targeting cognitive flexibility could be added to the Self-Regulation module for children who display poor cognitive flexibility. Such an enhanced program could also be adapted to teach educators strategies specifically designed to address children’s cognitive flexibility. As program implementation can be costly, integrating modules into existing programs for maltreated children could be easier and a more cost-efficient strategy than developing new programs.

Interventions in these environments are too often focused on the child’s behavior problems instead of on the reinforcement of their self-regulatory competencies (Chartrand et al., 2013). Studies have shown that caregivers play a significant role in the acquisition of emotion regulation competencies (Morris et al., 2007). Through modelling and reinforcement, the child learns to identify and regulate emotions properly. It has also been shown that cognitive flexibility is a factor of resilience that makes it easier to cope with change (Curran & Andersen, 2017). Furthermore, by allowing the child to exercise judgment, the educator encourages the development of cognitive flexibility. This is especially important for children in residential care who must adapt and respond to difficult situations such as conflicts with educators and peers daily (Chartrand et al., 2013).

## Conclusion

This study provides an initial understanding of the role of cognitive flexibility in the association between severity of child maltreatment and emotion regulation competencies in a sample of school-age children with severe early life adversities. Our results suggest that interventions aiming to enhance children’s cognitive flexibility could help prevent emotion regulation difficulties in those with less severe maltreatment experiences. For children with more severe experiences of maltreatment (higher number of maltreatment types, higher number of maltreatment notifications to CPS), more research is needed to understand the complexity of their deficits better and identify successful intervention targets. Nevertheless, the results of the present study are an important first step in advancing a field of research that is still relatively unexplored.
